# Hypersensitivity pneumonitis to phthalic anhydride: Case description and review of the literature 

**DOI:** 10.5414/ALX02519E

**Published:** 2024-08-19

**Authors:** Vera van Kampen, Anja Theile, Andrea Tannapfel, Christian Eisenhawer, Thomas Brüning, Rolf Merget

**Affiliations:** 1 Institute for Prevention and Occupational Medicine of the German Social Accident Insurance, Institute of the Ruhr University Bochum (IPA), and; 2 Institute of Pathology, Ruhr University Bochum, Bochum, Germany

**Keywords:** occupational hypersensitivity pneumonitis, plastics, phthalic anhydride, antibodies, diagnosis

## Abstract

Hypersensitivity pneumonitis (HP) is a rare, mostly occupational allergic disease of the lungs. There are many inhalable antigens that can cause HP. Most are organic dusts, rarely chemicals. A clinical case of HP is presented in a cable production worker with exposure to plasticizers who was initially diagnosed with idiopathic pulmonary fibrosis. The presence of specific IgG antibodies (sIgG) to phthalic anhydride in the patient’s serum, together with reduced carbon monoxide diffusion capacity, hypoxemia at rest and on exertion, and the findings on computed tomography and histology, seemed to confirm the diagnosis of chronic HP due to phthalates, particularly as exposure to phthalate compounds at work was reported by the Technical Inspection Service. A review of the literature revealed that there is evidence of plasticizer alveolitis. While in four previous case reports phthalic anhydride was suspected as the cause of occupational HP because of work-related symptoms, we were able to detect sIgG to phthalic anhydride for the first time. This case illustrates that phthalates, which have rarely been described as triggers of HP, should be considered in cases of suspected occupational HP.

## Introduction 

Hypersensitivity pneumonitis (HP) is a complex interstitial lung disease (ILD) resulting from an immune-mediated response in susceptible and sensitized individuals to a large variety of inhaled antigens, often through occupational exposures [1]. The antigens can be divided into predominantly organic substances (such as bacteria, fungi, animal, or plant proteins) and inorganic agents (such as low-molecular-weight (LMW) chemicals or metals). 

HP can be classified as acute/subacute or chronic. Patients with acute HP typically have non-specific respiratory symptoms such as cough, shortness of breath on exertion, fever, and general fatigue. Often, sudden flu-like symptoms occur within 4 – 12 hours after exposure. Patients with chronic HP, which is thought to result from long-term, low-level exposure, typically have a slowly progressive course. In this context, the gradual onset of non-specific pulmonary symptoms, such as cough and dyspnea, as well as fatigue and lack of or unnoticed acute episodes often leads to the misdiagnosis of other chronic ILDs, such as idiopathic pulmonary fibrosis (IPF) [1]. 

As mentioned before, HP is often caused by occupational exposure. According to Quirce et al. [2], the diagnosis of chronic occupational HP can be established if four or more of the following criteria are fulfilled: (i) exposure to a potentially offending antigen source at the workplace, (ii) elevated titer of specific IgG (precipitating) antibodies (sIgG) to an occupational antigen or bronchoalveolar lavage (BAL) lymphocytosis, (iii) reduced carbon monoxide (CO) transfer factor (DLCO) and/or hypoxemia at rest or exercise, (iv) high-resolution computed tomography (HRCT) pattern compatible with chronic HP, (v) pathology of lung specimen compatible with chronic HP, (vi) positive inhalation challenge test in the laboratory or positive workplace challenge or improvement after avoidance of the suspected occupational exposure. 

In most cases, HP is triggered by organic antigens, only rarely by chemicals [3]. In fact, according to a study of 116 patients with HP, the incidence was highest for bird-related disease (pigeon breeder’s/bird fancier’s disease) caused by exposure to feather dust and bird droppings followed by farmer’s lung and humidifier lung, both caused by exposure to bacteria and fungi [4]. Chemically induced HP is a rare phenomenon; however, some LMW substances that can trigger this disease have already been described. These belong to chemical groups such as diisocyanates, acrylates, metals, and acid anhydrides [2, 5]. 

One of the acid anhydrides is phthalic anhydride. It is known that phthalate compounds, presumably also phthalic anhydride, can be emitted during thermal processing of plastics at workplaces [6, 7, 8]. This is because most plastics contain plasticizers, mostly phthalate esters, which make them more flexible. It has long been known that phthalic anhydride can cause occupational asthma [9, 10], and in rare cases phthalic anhydride has been suggested as the putative antigen in occupational HP [11, 12, 13, 14]. However, in a literature review on chemical causes of occupational HP [15], none of these case reports was considered sufficient evidence that phthalic anhydride causes HP. 

A case of chronic HP in a cable production worker, formerly employed in a poultry farm, is presented here. 

## Case report 

### Occupational history, onset of symptoms, and previous findings 

Since 12/2001, the 59-year-old man had been employed as an operator in a cable factory. He was a former smoker and had stopped smoking 30 years ago. Regarding his previous employment, he worked in a poultry factory from 1983 to 1984, as a tractor operator in two companies from 1984 to 1992, and as a cleaner in a railway transport company from 1992 to 2001. In his current company, he first worked in the “industrial cables” department, and from 10/2017 he joined the “automotive cables” department where he developed his symptoms. Here he reported a higher temperature when processing plastic and different materials than in the “industrial #cables” department. 

According to the Technical Inspection Service of the responsible accident insurance, the patient was exposed to plasticizers in polyvinyl chloride (PVC) and rubber compounds since 2001. The formation of decomposition products could theoretically only occur in the event of temporary overheating of the raw material and complete emptying of the screw. However, a low exposure to phthalate esters and other phthalate compounds was reported. 

As early as 2017, he developed a recurrent cough and fatigue. The symptoms were clearly work-related; periods of sick leave led to improvement, but after returning to work, the symptoms rapidly worsened. After he additionally developed dyspnea on exertion in 2019, the first assessment of symptoms by a pneumologist was done in late 2020. Functional analysis indicated mild restriction with a total lung capacity (TLC) of 78% predicted (%pred.). The HRCT was interpreted as IPF. Systemic steroid medication was administered (40 mg/day). 

In early 2021, the patient was admitted to hospital with suspected acute HP. Lung function test showed a higher degree of restriction (no further information), and in BAL neutrophilia of 39% and lymphocytosis of 7% was detected. Initially, causality could not be established. However, following the detection of sIgG to pigeons/parakeets (no further information), causality was suspected, although the patient reported no pets or contact with animals and no use of bed feathers. 

Even one year after starting prednisolone therapy with 40 mg/day, there was no significant improvement in symptoms. For this reason, the patient was readmitted to hospital at the end of 2021. Lung function showed a higher degree of restriction (TLC 64%pred.). DLCO (41%pred.) and CO transfer coefficient (KCO, 71%pred.) were reduced. Lymphocytosis (no further information) and a CD4/CD8 ratio < 1 in BAL was reported. For histological confirmation of HP, two samples of peripheral alveolar lung tissue were obtained by video-assisted thoracoscopic surgery (VATS). In addition, the case of the patient was discussed by a multidisciplinary fibrosis board that recommended reducing the daily systemic steroid dose to 12.5 mg and starting treatment with nintedanib, a tyrosine kinase inhibitor (2 × 150 mg/day). 

### History, examinations, and medical findings at IPA (7/2022) 

The male patient (59 years, 1.75 m, 99 kg) came to our institute (IPA) in July 2022 for an examination within the scope of claims for compensation due to occupational disease. He gave written consent to the publication of all related data and images. Personal data, as well as both work and medical history were obtained by a questionnaire and physician interview. The patient had gained 25 kg over the last 2 years. He had not worked from March to June 2022, and there was a slight improvement in his symptoms. Prior to the examination he had again been unable to work for about 1 week due to worsening symptoms. He complained of exertional dyspnea and reported that he had to take a break after climbing 12 stair steps, his legs felt tired, and he had a lumpy feeling in his throat. He reported no flu-like symptoms. Medications were prednisolone 12.5 mg/day, and nintedanib 300 mg/day. On auscultation, fine crackles were heard bilaterally. Examination of the thoracic organs was otherwise unremarkable. 

Lung function was assessed by spirometry and body plethysmography with Power Cube Body (Ganshorn Schiller Group, Niederlauer, Germany) according to standard procedures [16] using reference values from the Global Lung Function Initiative (GLI) [17]. The results are presented in Table 1. The patient showed a decreased TLC (66%pred.). There was a reduction of forced expiratory volume in 1 second (FEV_1_) and forced vital capacity (FVC), but the FEV_1_/FVC ratio was normal (Table 1). In summary, lung function showed a moderate degree of restriction. 

DLCO was significantly reduced (57%pred.) and KCO was within the lower reference range (Table 1). During exercise on a cycle ergometer, blood gases were determined from hyperemic earlobe capillary blood. While there was a mild hypoxemia at rest, the partial pressure of oxygen in the blood (pO_2_) decreased from 66 to 57 mmHg after exercise with 50 W for 4 minutes. The exercise was terminated prematurely because of musculoskeletal complaints and dyspnea. In summary, the patient had both a reduced CO diffusing capacity and hypoxemia at rest and during exercise (Table 1). 

**Figure 1.**
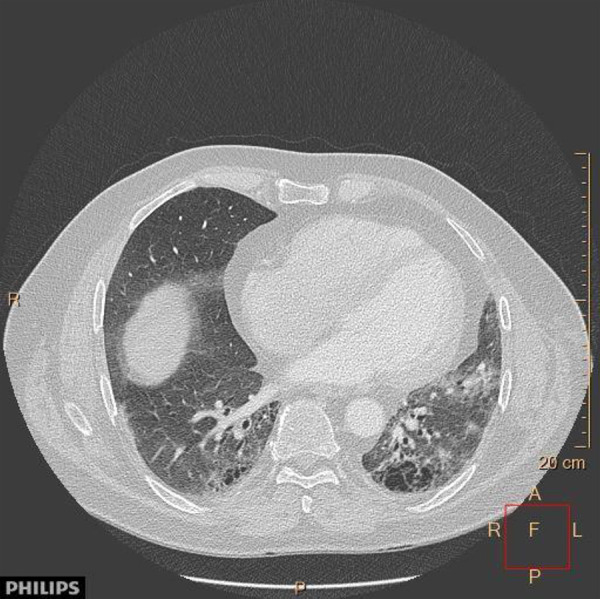
Computed tomography scan showing a florid inflammatory alveolar process with ubiquitous ground glass-like infiltrates in both lungs, cystic transformation, fibrotic changes, and septal thickening in the lower-lung zone with left-sided predominance and dilatation of the bronchi of both lower lobes.

Computed tomography (CT) of the thorax was performed after intravenous administration of contrast medium. There was a florid inflammatory alveolar process with ubiquitous ground glass infiltrates in both lungs. Cystic transformation of the apical lower lobe segment was seen bilaterally with left-sided predominance. There was marked dilatation of the dorsobasal bronchi (Figure 1). These findings are consistent with chronic HP and active areas. 

**Figure 2.**
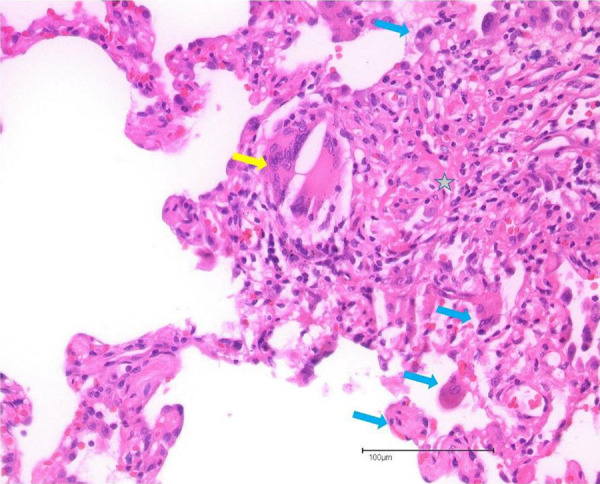
Histological specimens showing giant cells with cholesterol deposits (yellow arrow), multiple giant cells with inflammatory cells (blue arrows); lymphocytic intra-alveolar and interstitial inflammation with fibrosis (asterisk).

VATS had been carried out to confirm the diagnosis HP. Microscopically, a very representative peripheral alveolar lung tissue showed interstitial widening of the connective tissue with a dense lymphocytic-lymphohistiocytic follicular inflammatory cell infiltrate. There were disorganized loose histiocytic granulomas with multinucleated giant cells. Subpleural foci showed a confluence of connective tissue foci and abortive fibroblast proliferation in a cushion-like myxoid architecture protruding against the intact lung parenchyma (Figure 2). In summary, a high grade chronic and highly florid lymphohistiocytic fibrosing alveolitis was seen which, in combination with the characteristic loose giant cells with cholesterol clefts, was highly supportive of chronic HP. 

Total IgE as well as specific IgE antibodies (sIgE) and sIgG were measured using the ImmunoCAP system (ThermoFisher Scientific, Phadia AB, Uppsala, Sweden). Total IgE levels < 100 kU/L were defined as normal. For specific antibodies, positivity criteria were sIgE concentrations ≥ 0.35 kU/L and sIgG concentrations above the antigen-specific reference values. Values above the 97.5% quantile of a control group were classified as elevated [19]. Total IgE was normal (6.1 kU/L), and sIgE to acid anhydrides were negative (Table 1). 

Concentrations of sIgG to the mold *Aureobasidium pullulans*, to the bacterium *Saccharopolyspora rectivirgula*, as well as to chicken and goose feathers and phthalic anhydride were elevated compared with reference values (Table 2). It is important to note that sIgG binding to LMW phthalic anhydride was tested using a conjugate of phthalic anhydride and human serum albumin (HSA), so the extent of non-specific IgG binding to HSA must also be considered. Although this was elevated, it was below the 97.5% quantile cut-off (Table 2). 

In addition to the clear work-related symptoms, the elevated concentration of slgG to phthalic anhydride is indicative of the origin of the disease. Taking into account the criteria for the diagnosis of chronic occupational HP mentioned in the introduction, all of them are met: (i) exposure to phthalate compounds at the workplace, (ii) elevated concentration of slgG to phthalic anhydride, (iii) reduced CO diffusing capacity and hypoxemia at rest and during exercise, (iv) CT pattern compatible with chronic HP, (v) pathology of lung specimen compatible with chronic HP, (vi) (slight) improvement of symptoms during periods of sick leave. 

As there is also evidence of plasticizer alveolitis in the medical literature (see below), the final diagnosis was chronic occupational HP due to phthalic anhydride. 

### Review of the literature for HP due to phthalic anhydride 

A systematic search of international studies listed in PubMed (MEDLINE) using the following MeSH (Medical Subject Headings) terms # hypersensitivity pneumonitis OR (extrinsic allergic) alveolitis AND phthalic anhydride OR acid anhydride OR plasticizer OR polyester. Moreover, we checked references in papers previously identified and added relevant studies (snowballing). Only papers in English and German language were included. 

Although no cases of HP have been reported, a publication from 1955 already contains some reports suggesting that alveolitis may be involved in respiratory disease caused by phthalic anhydride exposure [20]. Another publication on HP in general also mentions acid anhydrides and specifically phthalic anhydride as rare possible triggers of HP [21]. In addition, there are four published case reports of HP associated with plastics and plasticizers and possibly with phthalic anhydride. 

Cartier et al. [11] described the case of a 39-year-old man who developed symptoms of both asthma and HP after being exposed to polyester powder paint containing different terephthalates. The paint was sprayed onto the metallic boards, which were then heated at 200 °C. About 3 weeks after starting his work next to the oven, he developed symptoms of coughing, shortness of breath, sweating, and shivers at the end of a shift. The symptoms lasted for 3 – 4 hours in the evening. He had no respiratory problems before starting work. For a month the symptoms recurred, but not every day and never at weekends when he was asymptomatic. The asthmatic component of the disease was confirmed by a significant reduction in FEV_1_ immediately after specific inhalation challenge (SIC) with heated polyester. However, the patient also had fever, leukocytosis, and a reduced DLCO, which the authors interpreted as an alveolitis-type reaction. They ruled out trimellitic anhydride (TMA), which like phthalic anhydride can be released when polyester is heated, as the cause by performing a SIC with TMA, which was negative. Furthermore, sIgE and sIgG to TMA coupled to HSA were within normal limits. The authors speculated that the alveolitic reaction was due to released LMW compounds such as aldehydes. 

In the second case report, polyester powder paint was also suspected as the cause of HP, and it should be clarified whether phthalic anhydride or TMA is the alveolitis-causing agent [12]. Both were found at low levels (< 1%) in the paint used occupationally by a 61-year-old woman with recurrent chills, cough, shortness of breath, and fever. Symptoms decreased during sick leave and holidays. She had been exposed to dust and fumes during the painting and curing process (180 – 240 °C) for ~ 8 years prior to the onset of symptoms. Crackles were heard on pulmonary auscultation on the right side and blood leukocytosis was found. On chest radiography, thin atelectases were noted. Returning to work, she again experienced symptoms and fever (39.2 °C). Peak expiratory flow decreased from 440 to 240 L/min during the working day. Spirometry showed a restriction (FEV_1_ 56%pred., FVC 54%pred.). Two months later, a BAL was performed showing lymphocytosis (67%) and a low T-helper/T-suppressor ratio (0.2). However, serum sIgG to phthalic anhydride and TMA, measured using the enzyme-linked immunosorbent assay technique, were negative. During SIC with heated polyester powder paint, FEV_1_ decreased by 14% and DLCO decreased by 8%. Air samples were taken during the SIC; while no method was available to measure TMA, a concentration of 0.054 mg/m^3^ was found for phthalic anhydride. The authors discuss that their case report has similarities with the case described above by Cartier et al. [11], and that phthalic anhydride and TMA should be considered as potential causative agents for HP. 

Volkman et al. [13] reported a case of HP in a 46-year-old woman working at a yacht manufacturing company, where she was rolling out wet sheets of polyester resin-impregnated fiberglass, which were then placed in a mold for curing. She reported a 2-month history of work-related progressive dyspnea, chest tightness, and daytime, nocturnal, and exertional cough. Treatment with systemic antibiotic therapy, inhaled bronchodilators, and inhaled corticosteroids provided minimal relief of symptoms. A chest X-ray showed a diffuse interstitial pattern. Spirometry showed a restriction (FEV_1_ 41%pred., FVC 43%pred.), but lung function improved with oral corticosteroids and with avoidance of work. However, re-exposure at work after 6 weeks again resulted in a decrease in both lung function parameters, and DLCO was also consistent with a restrictive ventilatory defect. Her symptoms progressed to exertional dyspnea and nocturnal cough with continued occupational exposure. A CT of the chest demonstrated nodular densities, and a blood count showed 77% neutrophils, 16% lymphocytes, and 7% monocytes. The authors considered the history and objective findings to be consistent with occupational HP. Although the specific chemical or antigen could not be determined, dimethyl phthalate and styrene were considered the most likely causative agents. According to the authors, a literature search found no scientific literature confirming styrene as a cause of HP. 

In a recent publication, Sartorelli et al. [14] reported the case of occupational HP in a 66-year-old male non-smoker who had worked for more than 20 years in a chemical company producing polyethylene terephthalate (PET) for disposable beverage bottles. Prior to working in PET production, he had worked as a mechanic in a ship’s engine room and had been exposed to asbestos through numerous repairs. At his first medical examination, he was diagnosed with asbestosis and pleural plaques. During the follow-up as an asbestos-exposed worker, HRCT showed a slight progression of the pulmonary fibrosis not only in the extension but also in the profusion of CT features, both in the upper and lower lobes. In the meantime, the patient had developed progressive dyspnea. On auscultation, inspiratory crackles were heard at the lung bases, and lung function showed a decreased DLCO. Moreover, BAL analysis showed 71% macrophages, 14% lymphocytes, 6% neutrophils, 9% eosinophils, and a CD4/CD8 ratio of 1.68. Histological examination of lung biopsies revealed bronchiolocentric lymphocytic interstitial pneumonitis with scattered eosinophils and an organizing pneumonia pattern. Rare and poorly formed interstitial granulomas were present. In the most advanced areas, destructive scarring with architectural distortion, but with centrilobular and periseptal prevalence were seen. No asbestos bodies were detected. Therefore, the diagnosis of HP was made and linked to occupational exposure to terephthalic acid or dimethyl terephthalate. 

## Discussion 

As our literature review showed, there are references to plasticizer alveolitis in the medical literature, which we were able to confirm in the case presented. In contrast to the four case reports shown above, in which phthalic anhydride was suggested to be the cause of occupational HP, we were able to detect sIgG to phthalic anhydride for the first time. 

An overlap between IPF and fibrotic HP has been reported in the literature [1]. In a case-cohort study, 20 of 46 (43%) patients with IPF had a subsequent diagnosis of chronic HP mostly based on surgical lung biopsy [22]. Our patient was also previously diagnosed with IPF before the final diagnosis of chronic HP was confirmed histologically. Although steroids are no longer recommended for the treatment of IPF, systemic steroid therapy was initiated prior to the final diagnosis of chronic HP, possibly while steroids are still used to reduce cough [23]. 

An international modified Delphi survey shows the importance of proven exposure for the diagnosis of HP. Two different case scenarios emerged for the definitive diagnosis of chronic HP: first, an identified exposure in the history combined with HRCT features suggestive of chronic HP and a lymphocyte count > 40% of total cells in BAL; second, any scenario with an identified exposure and a lung biopsy with features of chronic HP. None of the scenarios with no exposure lead to a definitive diagnosis, even after including histopathological features [24]. 

Unfortunately, we had only limited information on the patient’s two BAL examinations. The absence of lymphocytosis in the BAL at the beginning of 2021 may be due to high antigen exposure shortly before. In this context, Quirce et al. [2] stated that the absence of lymphocytosis in BAL makes the diagnosis of HP unlikely but is possible within the first 48 hours after intense antigen exposure. In fact, the BAL examination was performed when the patient was hospitalized on a Tuesday for 2 days due to suspected acute HP. However, it can only be assumed that the hospitalization was preceded by an intense antigen exposure. Furthermore, unlike acute/subacute HP, the role of alveolar lymphocytosis in the diagnosis of chronic HP is still unclear [25]. The meta-analysis showed that the degree of pulmonary fibrosis, the type of antigen and the time since last exposure influence the degree of alveolar inflammation in HP, with a lower lymphocyte percentage in chronic than in acute HP. In addition, older age and a history of smoking were also associated with a lower lymphocyte percentage [25]. 

As an identified exposure is essential for the definitive diagnosis of HP [24], the fact that we were able to detect sIgG to phthalic anhydride supported our diagnosis of plasticizer alveolitis. Although the patient also had sIgG to bacteria, molds, and birds, the latter in high concentrations, only phthalic anhydride exposure was causally and temporally related to his symptoms. In our patient, we were unable to confirm any evidence of alveolitis due to avian proteins, which, according to the literature, cause about half of chronic HP cases [1]. His former work in a chicken breeding factory cannot be seriously discussed due to the long latency, the patient never kept birds in his private life, and there was no exposure to bed feathers. In particular, non-occupational feather contact (e.g., with down products) was questioned in detail, but credibly denied. 

Since the medical history, the known exposure to phthalate compounds and especially the exposure-related symptoms tend to favor phthalic anhydride as the responsible antigen, we consider it more likely that HP in our patient is occupational than non-occupational. In addition, according to the literature and our many years of experience, a positive result for sIgG binding to the phthalic anhydride-HSA conjugate is extremely rare. Considering the study on sIgG reference values for HP antigens [19], the relatively high non-specific binding to HSA does not call this into question. In this study, low sIgG levels were found against the tested chemicals, which were highly positively correlated with HSA-sIgG levels. Based on all data, the authors classified HSA-sIgG levels < 4 mgA/L as non-specific [19]. It is known that HP patients often are sensitized to additional antigens in the further course of their disease [26]. This may explain why our patient had sIgG against several antigens. In general, the presence of sIgG reflects an immune response to a specific exposure, indicating that the individual had a sufficient level of exposure to the antigen to develop sensitization. However, this alone is not sufficient to establish the diagnosis, as many asymptomatic individuals have a similar level of humoral response [5]. 

To our knowledge, there is only one case where sIgG was detected against another acid anhydride (pyromellitic acid dianhydride) in an epoxy resin production worker clinically diagnosed with acute hemorrhagic alveolitis [27]. 

An analysis of the peer-reviewed literature on HP and chemicals showed the expected low level of LMW chemicals identified in case reports as causing occupational HP [15]. HP is a much rarer disease than occupational asthma, and LMW chemicals are much less frequently reported as causative agents of HP than high-molecular-weight substances (e.g., moldy hay, bird proteins). 

If left untreated, HP can lead to progressive pulmonary fibrosis with associated morbidity and mortality. The cornerstone of treatment is early elimination of exposure to the causative antigen, although the disease may take an unfavorable course even if exposure to the causative agent is avoided. Unfortunately, this was the case with our patient. When contacted in February 2024, he reported a slight worsening of his symptoms despite continued exposure cessation. If an employee is diagnosed with occupational HP in a company, colleagues in the same work area should be examined immediately. This will allow early identification and treatment of other potentially affected or ill workers. Appropriate preventive measures should also be taken to ensure health and safety in the workplace [28]. 

As many cases of HP are caused by occupational exposures, a detailed occupational history should be taken in all patients with suspected HP based on history or imaging. As our case illustrates, also the rarer LWM antigens, especially phthalates, should also be considered as triggers of occupational HP. 

## Acknowledgment 

Special thanks to the patient for allowing us to publish his case. 

## Authors’ contributions 

Investigation: RM, ATh, AT - Data Curation: VvK, RM; Data Interpretation: RM, VvK, ATh, AT, CE, TB; Writing (Original Draft): VvK; Writing (Review & Editing): RM, ATh, AT, CE; Supervision: TB. All authors read and approved the final manuscript. 

## Funding 

This research did not receive any specific grant from funding agencies in the public, commercial, or not-for-profit sectors. The data were collected as part of investigation within the scope of claims for compensation due to occupational asthma at the Institute for Prevention and Occupational Medicine, Institute of the Ruhr University Bochum (IPA) which is financed by the German Social Accident Insurance (DGUV). 

## Conflict of interest 

All authors declare no conflict of interest. The authors VvK, CE, TB, and RM are employees of IPA. They are independent from the DGUV in access to the collected data, responsibility for data analysis and interpretation, and the right to publish. 

**Table 1.** Characteristics, symptoms, and test results of the patient with suspected occupational hypersensitivity pneumonitis at the time of examination in our outpatient clinic.

	**Case**
Sex, age (years)	Male, 59
Smoking status	Former (30 years ago)
Suspected allergen	Phthalate compounds
Work-related symptoms	Cough, fatigue, dyspnea
Duration of symptoms (years)	5
Lung function test	
RV %pred. [%] (z-score*)	77 (–1.3)
TLC %pred. [%] (z-score)	66 (–3.4)
RV%TLC [%] (z-score)	108 (0.6)
FEV_1%_pred. [%] (z-score)	58 (–2.7)
FVC %pred. [%] (z-score)	59 (–2.9)
FEV_1_/FVC [%] (z-score)	100 (0.0)
Diffusing capacity	
DLCO %pred. [%]	57
KCO %pred. [%]	89
Blood gases (ergometry) – at rest:	
pO_2_ (mmHg) (%pred. [%])	66 (81)
pCO_2_ (mmHg) (%pred. [%])	37 (94)
– on exercise (50 W):	
pO_2_ (mmHg)	57
pCO_2_ (mmHg)	42
Total IgE (kU/L)	6.1
Specific IgE (sIgE) to (kU/L)	
Phthalic anhydride (k79)	0.00
Maleic anhydride (Rk210)	0.00

FEV_1_ = forced expiratory volume in 1 second; FVC = forced vital capacity; KCO = carbon monoxide coefficient; pO_2_ = partial pressure of oxygen in the blood; pCO_2_ = partial pressure of carbon dioxide in the blood; RV = residual volume; TLC = total lung capacity; DLCO = transfer factor of the lung for carbon monoxide. *A z-score greater than −1.645 is considered normal [18].

**Table 2.** Results of specific IgG antibody testing and evaluation of test results according to [19].

**Antigen**	**mgA/L**	**Rating**	**95% quantile of 121 references**
*Aspergillus fumigatus*	22.93	n.e. (52.3% quantile)	97.22
***Aureobasidium pullulans***	22.84	**high (98.8% quantile)**	12.28
*Penicillium chrysogenum*	18.29	n.e. (52.6% quantile)	71.55
Mould mixture	17.44	n.e. (81.7% quantile)	26.80
***Saccharopolyspora rectivirgula***	10.95	**high (98.8% quantile)**	5.17
*Thermoactinomyces vulgaris*	15.95	n.e. (88.1% quantile)	21.46
**Chicken feathers**	35.71	**higher than max. control**	12.62
**Goose feathers**	26.73	**higher than max. control**	11.54
**Phthalic anhydride**	5.89	**high (97.7% quantile)**	3.70
Human serum albumin (HSA)	2.93	n.e. (95.4% quantile)	2.74

Antigens for which the patient had sIgG values above the 97.5% quantile of the reference group are shown in bold. n.e. = not elevated.
